# Metropolitan Hospital Sunday Fund

**Published:** 1895-07-06

**Authors:** 


### The Metropolitan Hospital Sunday Fund.

SECOND LIST OF DONATIONS.

Cheques continue to be sent to the Lord Mayor and the Secretary at the Mansion House. Lord Iveagh and Sir Savile Crossley have each sent a donation of £1,000. We have reason to hope that these seasonable gifts will be followed by others before the Fund is finally closed, the large brewing firms, including Messrs. Guinness, Messrs. Allsopp, Messrs. Reid, and the Lion Brewery having sent between them £1,350. This is a good beginning, and we could wish their example may be followed not only by other brewers, but by leading teetotallers whose names are at present conspicuous by their absence. Altogether, the contributions received exceed £38,000, of which about £5,000 represents donations apart from Church collections.

**Donations of £1,000.**

Sir Savile Crossley. Lord Iveagh.

**Donation of £200.**

"Delta" (a further).

**Donation of £52 10s.**

R. W. Inglis.

**Donation of £25.**

A. Miller (a further).

**Donation of £21.**

C. H. T. Hawkins (a further).

**Donations of £20.**

W. M. Cross (a further). W. T. Blanford (a further).

**Donation of £10 10s.**

Miss Nob'e (a further).

**Donations of £10.**

A. F. Williams (a further). Frank Newson (a further).

Edward L. Benyon (a further). Lord Stratheden.

Miss M. E. Druce (a further).

**Donations of £5 5s.**

Rev. Francis Palmer (a further). C. H. Fox.

F. E. Hills. A. S. Churchill (a further).

Henry Bull.

**Donation of £5 1s.**

Principals, Clerks, and Workmen of J. W. Duffield and Sons, builders, Kensington, W.

**Donations of £5.**

Mrs. Longsdon (a further). Lieut.-Colonel Boyd Alexander (a further).

Mr. Longsdon (a further).

A. E. F. Horniman (a further). G. F. Findlay (a further).

Mrs. Henry Bentinck (a further). G. W. Dennis (a further).

Mrs. Ramsay L'Amy (a further). J. Macfadyen.

Henry Farmer.

**Donation of £3 5s.**

C. H. Heward, Son, and employés (a further).

**Donations of £3 3s.**

T. H. Lee. S. Strickland.

**Donations of £3.**

A. Boyd (a further). E. C. Smith.

**Donations of £2 2s.**

L. T. Topham. A. C. Bishop (a further).

J. J. Sylvester (a further). Vicars Boyle.

J. Roberts.

**Donations of £2.**

Mrs. Hodgson. M. G. Duncan.
Mrs. Bullen Smith. Mr. Jackson.
Dr. Bramley (a further). A. Garrard.
George Hall.

**Donation of £1 13s. 9d.**

United Asbestos Company, Fairfield.

**Donation of £1 2s.**

A. J. Rudkin.

**Donations of £1 1s.**

F. G. Bennett. John Scoble.
W. G. Fossick. H. S. Frower.
T. W. Evans (a further).

**Donations of £1.**

W. Davisson. John Power.

Miss Rosalind Pritchard. Albert Anns.

G. Williams (a further). E. J. Stephens.

C. O. Murray. H. C. Trollope.

Anonymous donations and contributions below £1 hitherto unacknowledged amount to £30.

